# Gait Speed with Anti-Slip Devices on Icy Pedestrian Crossings Relate to Perceived Fall-Risk and Balance

**DOI:** 10.3390/ijerph16142451

**Published:** 2019-07-10

**Authors:** Agneta Larsson, Glenn Berggård, Peter Rosander, Gunvor Gard

**Affiliations:** 1 Department of Health Sciences, Luleå University of Technology, Luleå 97187, Sweden; 2 Department of Civil, Environmental and Natural Resources Engineering, Luleå University of Technology, Luleå 97187, Sweden

**Keywords:** anti-slip device, classification, postural control, pedestrian crossing, safety, gait speed, winter conditions

## Abstract

It is important to find criteria for preventive measures and appropriate assistive devices to reduce pedestrian injuries and increase walking in winter. Reducing the rate of falls on icy surfaces and improving people’s ability to safely cross a street in winter conditions by achieving an adequate walking speed, for example, need to be considered. This study explores pedestrian perceptions of fall risk, balance, and footfall transitions while using different designs for anti-slip devices on ice and snow-covered ice and relates these to measures of gait speed and friction. Trials were performed with nine pedestrians testing 19 anti-slip devices on ice and ice covered with snow. Laboratory tests of the dynamic coefficient of friction (DCOF) on plain ice were also performed. The findings suggest that there was conformity in the participants’ perceptions of good balance and low fall risk for one-fifth of the devices (three whole-foot designs and one design with built-in spikes). We also found that gait speed on icy pedestrian crossings is related to perceived fall-risk and balance control, but not to DCOF of the anti-slip devices.

## 1. Introduction

Slip-induced falls in winter are a substantial cause of pedestrian injuries [1,2,3], and the costs for medical care of fall-related injury treatment are high [4]. In Arctic regions, pedestrians encounter a range of environments and pavement or road surface conditions, such as snow, ice, melting ice, or mixed icy and snowy surfaces [5,6]. Evolving climate change, causing warmer winters, reduced snow cover, and increased rainfall [7] could also influence pedestrian safety related to surface conditions. It has recently been shown that other risks have emerged than those normally considered in sub-arctic areas, and rain, icy surfaces, and darkness are today’s most significant barriers to community walking. Unknown conditions entail the risk based on anticipated weather and surface conditions not being reliable [8]. 

A slip may occur when the required friction between the shoe and the ground surface is insufficient [9]. One method for improving the friction is to attach anti-slip devices on a person’s ordinary shoes. Anti-slip devices are acknowledged to be personal safety equipment, and CE (Conformité Européenne) certification is required. A CE (Conformité Européenne) marking declares that the product fulfils the applicable European Union health, safety, and environmental protection requirements, and can be sold within the European economic area. 

Another aspect to consider is the capability of the human postural control system to recover from slip perturbations [10,11]. Postural control is a complex motor skill based on the interaction of multiple sensorimotor processes to maintain a state of equilibrium and postural orientation. It involves the ability to control the body’s centre of mass in relation to the base of support during both voluntary movement and when being exposed to external perturbations [12] such as slipping. Unexpected slip disruptions during gait tests reactive responses, and response during early stance phase is especially critical for reactive balance recovery [13]. In contrast, anticipation of a potentially slippery surface brings anticipatory postural adjustments, including changes in the activation of lower-limb muscles and hip and knee joints [14,15]. Increased knowledge of mechanical friction measurements, as well as human-centred methodologies for measurement of slips and balance recovery, is essential for the prevention of fall injuries [10,13]. Whilst community walking is required in a wide range of environmental conditions [16], test methods that contain the complex walking tasks encountered in daily life, such as changing speed and direction [17], are useful in helping to evaluate preventive measures and assistive devices. It is plausible that appropriate shoes and anti-slip devices, in addition to reducing the rate of falls on icy surfaces [18], also improve people’s ability to cross a street in winter conditions safely. Earlier research has found that adequate walking speed at a pedestrian crossing is crucial for pedestrian safety. However, fast walking speed is not possible for everyone in the population, even in summer conditions [19]. Another plausible benefit with enabling people to feel safe when walking in winter is the prospect of more frequent use of non-motorised modes of transport [6] and obtaining the general health benefits of physical activity [20]. Seasonal patterns of decreased physical activity in winter have been reported, especially among middle-aged adults [21]. 

This study explores pedestrian perceptions of fall risk, balance, and footfall transitions while using different designs for anti-slip devices on ice and snow-covered ice and relates these to measures of gait speed and friction. The research questions were: 

1. How did the pedestrians rate their fall risk, balance, heel strike, and toe off on plain ice and snow-covered ice in the different anti-slip devices? 

2. How were the measures of gait speed related to ratings of fall risk and balance and to the dynamic coefficients of friction (DCOF) of the devices?

## 2. Materials and Methods 

This research was conducted within a larger project for the development of test methods and criteria for efficient anti-slip devices [22]. The research was performed in two experimental settings. The dynamic coefficient of friction (DCOF) of the devices were tested in a laboratory at the Finnish Institute of Occupational Health (FIOH) and at an indoor ice rink where realistic pedestrian crossing situations were simulated on various walkway surfaces (tracks) of ice, snow, and concrete. To reach a deeper understanding of changes in the study participants’ gait patterns and perceptions during the trials, a combination of subjective ratings and objective measures were used. In total, nine study participants tested 19 anti-slip devices.

### 2.1. Anti-Slip Devices Tested

Nineteen anti-slip devices, reflecting various designs, were selected for testing. The devices were classified into four design groups. The first three groups were devices attached to the subjects’ shoes: 1) A forefoot device that covers only the front of the sole (three devices/2–3 studs); 2) a heel device that covers only the rear of the sole (three devices/4–5 studs); 3) a whole-foot device that covers the entire sole (eight devices: 6–12 spikes; three devices: Sand/rope/springs). The fourth group were two shoes with different designs for built-in studs (one solution with 16 retracting studs attached to the sole; one fixed and foldable solution with 20 studs). 

**Figure 1:**
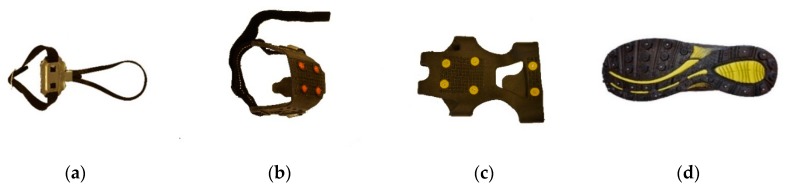
Examples of the main type models: (**a**) Forefoot device, #1; (**b**) heel device, # 4; (**c**) whole-foot device, #13; and (**d**) shoe with built in studs, #19.

Most devices are made of different polymers that hold together the construction and metal as friction-increasing material (except that with sandpaper) and are available in small, medium, and large sizes (Table 1 and Figure 1). 

### 2.2. Measures of DCOF

The International Organisation for Standardisation (ISO) is a worldwide coalition of national standards organisations. An ISO document is developed as an international standard, and an EN preface to the document indicates that it is intended to be used in the European Union.

In this experiment, the DCOF of the devices were tested in ambient laboratory conditions at FIOH using a method amended from EN ISO 13287:2012, which is the standard for safety shoes. FIOH further developed the method to suite conditions with ice and anti-slip devices or shoes with built-in spikes.

The air temperature in the laboratory was +21 °C. Devices were tested on a smooth ice surface (5 mm thick), −4.6 to −5.4 °C, and the mean hardness of the ice was 13.54 MPa. The hardness corresponds to natural outdoor ice [23] and was harder than the indoor ice (9.36 MPa) at the experimental set-up at the ice rink. A colder temperature implies harder ice [23].

**Figure 2:**
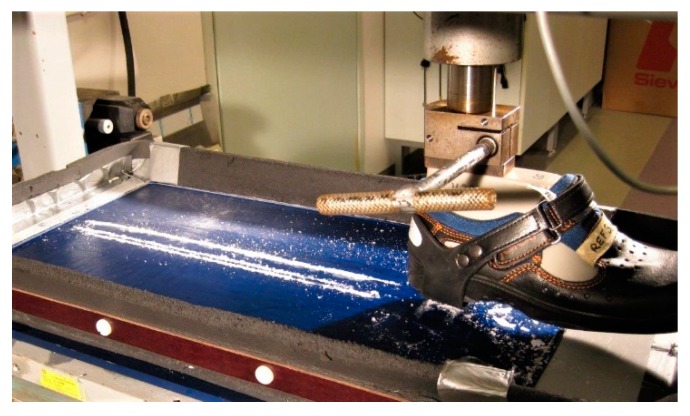
The dynamic coefficient of friction (DCOF) measuring method.

The devices were tested in random order. Each device was mounted to a reference shoe on an artificial foot, and 12 tests in a row were performed per device. Mean and standard deviations were calculated (Scale 0−1.00). Each test was performed on a new ice surface: 1) A normal force of 500 ± 25 N was applied on the ice surface through a reference shoe (UK size 6.5, make: Jalas) and attached device, at a 90° angle to the surface; 2) sliding the device parallel to the ice in a forward direction was performed with a sliding velocity of 0.3 (±0.03) m/s. The frictional force was measured against the direction of movement (Figure 2). 

### 2.3. Experimental Set-Up

#### 2.3.1. Test Tracks

**Figure 3:**
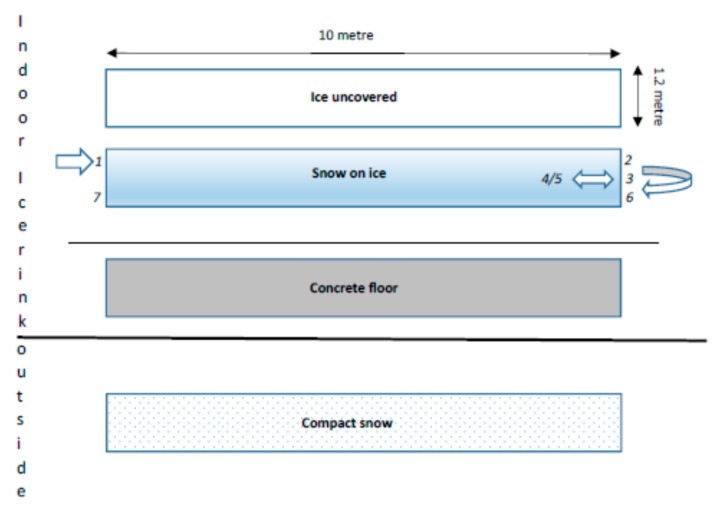
Sketch of the test tracks.

Four test tracks were prepared at the indoor ice rink, each one 10 metres long and 1.2 metres wide: Plain ice, ice covered with 3–5 mm snow, a dry concrete floor, and a compact snow track outside the ice rink. The devices were tested in random order and according to a procedure developed in earlier experiments [3]. Each participant walked with all anti-slip devices on each surface according to the following instructions: 1) Walk at a comfortable speed along the track, 2) turn around, 3) walk rapidly two steps, 4) stop, 5) walk backwards two steps, and 6) walk rapidly along the track; starting at the line and keeping the walking speed until having crossed the end line of the track (Figure 3). The aim was to simulate a realistic, crosswalk situation. By this procedure, strain was induced on the devices through different transitions and forces.

#### 2.3.2. Preconditions 

In this study, data from the trials on plain ice and snow-covered ice are analysed. The experiment was performed over four days. The hardness of the smooth ice surface was tested, and temperature and humidity were measured on the ice surface and two metres above the ice surface. Measures on the four days showed minor variations in hardness, with mean values of 9.36 MPa for the indoor ice. The air temperature on the surface of the ice varied by 1.5 °C (mean −3.9 °C), which was considered not to have an impact on the results. The indoor air temperature varied by 2.1 °C (mean 4.9 °C). There were smaller variations in humidity on the ice surface than in the air. The degree of humidity on the ice can affect how the snow attaches to the ice. 

#### 2.3.3. Participants

The participants, five women and four men, with a mean age of 47 (41–71) years, corresponded to a normal population in terms of variations in background parameters, previously described in detail [22]. Of these, seven subjects had previously experienced an outdoor fall accident, and five had previous experience using anti-slip devices. There were great variations between the participants’ perceived risk of falling (Md 69, range 4–95) and balance control (Md 78, range 2–96) during outdoor walking in wintertime, rated on a scale 0–100.

The study was performed in compliance with the ethical principles for good research practice. This includes that informed consent was obtained from each participant. Risks involved were managed by protective equipment (kneepads and helmet) provided, and participants were encouraged not to expose themselves to unnecessary risks or exceed their ability during the trials on ice tracks. 

### 2.4. Measurements 

#### 2.4.1. Background Gait Parameters of Participants

Participants’ indoor gait patterns were registered by a 10-metre timed walking test at a comfortable speed and maximum speed, respectively [24]. To evaluate the base of support, footfall transitions, speed, step length, rhythm, and single- and double-support phases during the gait cycle, subjects walked with and without shoes on a 4-metre long digital carpet with embedded pressure sensitive switches, GAITRite®. It is connected to a personal computer, and temporal and spatial parameters of gait are automatically calculated after the subject completes a trial [25,26]. 

Baseline indoor gait speed, as well as stride length and double-support phase, varied between participants (Table 2).

#### 2.4.2. During the Experiment

During the trials, participants were asked to rate different qualities of the gait cycle, including heel strike, toe off, balance, and risk of falling, on a VAS scale (0−100) [27] for each anti-slip device on each surface. The results were registered in a protocol. Also, while using each anti-slip devices on each surface, each participant’s comfortable (between points 1 and 2 in the test sequence, see Figure 3) and rapid gait speed (between 6 and 7), respectively, was timed (seconds) by the test leaders and registered in the protocol. Gait speed (cm/sec) was calculated. 

### 2.5. Data Analysis 

Baseline data on gait were analysed with GAITRite®. IBM® SPSS® Statistics 25 (IBM Corporation, Armonk, NY, USA) was used to analyse data on time and participants’ ratings on scales, and these are presented with a median (max and min) value. Mean and standard deviation measures were used to show the DCOF. 

To identify significant qualities of anti-slip devices, we defined ‘good walking balance’ as ≥80 points and ‘low fall risk’ as ≤20 points on a 100-point scale. Participants matching ratings for the impact of the device on balance and fall-risk was defined as inter-subject variations ≤30 points on a 100-point scale. A ‘higher maximum gait speed’ was defined as ≥160 cm/sec. Wilcoxon signed ranks test was used to assess the difference between two measurements on a single sample. 

## 3. Results

### 3.1. Pedestrian Ratings of Fall Risk, Balance, and Footfall Transitions on Uncovered Ice and Snow-Covered Ice with Different Anti-Slip Devices 

**Figure 4:**
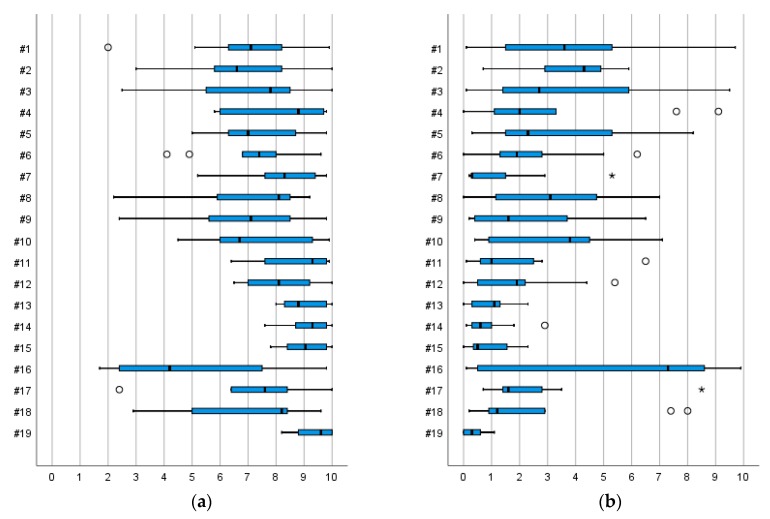
(**a**) Perceptions of walking balance and (**b**) of risk of falling on an uncovered ice surface, respectively, with the 19 devices. Scale 0–100. Boxplot graph shows median values and upper and lower quartiles. Whiskers indicate variability outside the quartiles and outliers are plotted as individual points.

We tested four main designs of anti-slip devices, namely forefoot devices, heel devices, whole-foot devices, and devices with built-in spikes. In general, there was little consensus among participants in their perceptions of fall risk, balance, heel strike, or toe off when walking with the devices on ice, with a few exceptions. With nine devices, the participants perceived both low fall risk (defined as ≤20 points) and a good walking balance (≥80 points) on uncovered ice. For four of these—whole-foot devices #13, #14, and #15 and one in-built device, #19—the participants agreed in their perceptions (defined as inter-subject variation ≤30 points on a 100-point scale) of balance *and* fall risk, respectively. Another five devices—heel device #4, whole-foot devices #7, #11, and #12, and in-built spikes #18—received good ratings concerning fall risk and walking balance, although the participants’ perceptions varied more (Table 3 and Figure 4).

On the snow-covered ice, 12 devices received good ratings for providing low fall risk and good walking balance. Shared views were shown for three devices—whole-foot devices #14, #15 (sand) and built-in spikes, #19—for good walking balance, and for one device—the whole-foot device #11—in terms of low fall risk (Table 4 and Figure 4). 

### 3.2. Gait Speed, DCOF, and the Relations of Pedestrian Ratings to these Measures 

The participants tended to walk a bit slower on the ice track with most devices (Table 3) in comparison to their comfortable gait speed at baseline (Md 139.3 cm/sec) (Table 2). This decline in comfortable speed on ice was statistically significant (*p* < 0.05) for devices #2, #8, #9, #12, #16, and #17. On snow covered ice, the speed was significantly reduced for device #2. 

**Figure 5:**
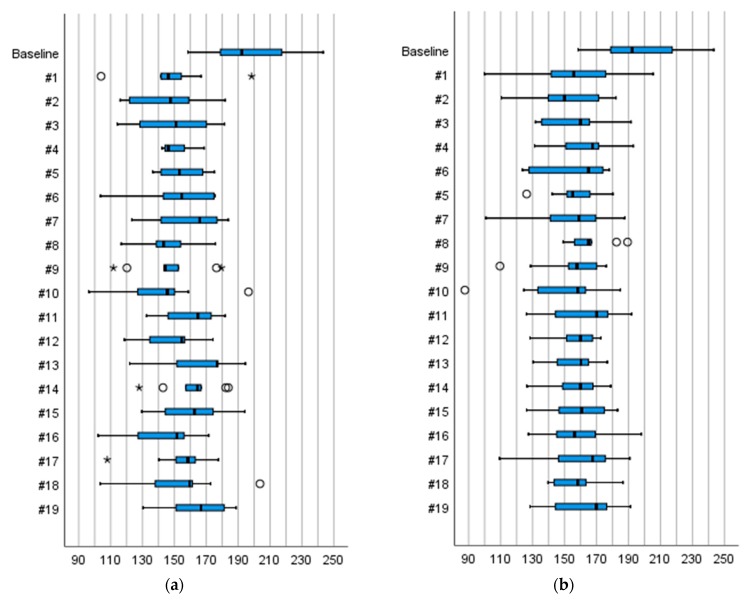
(**a**) Maximum gait speed (cm/sec) on uncovered ice in comparison to baseline speed (without the device), and (**b**) maximum gait speed on snow-covered ice.

Their ability to reach their maximum gait speed when asked to walk as fast as possible was significantly reduced in the experimental situation with all 19 devices on ice (Table 3 and Figure 5) and snow-covered ice (Table 4 and Figure 5) in comparison to gait speed (Md 192.3 cm/sec) at baseline (Table 2).

There seems to be a pattern that six (#7 #11, #13–15, #19) of the nine devices with low fall risk/good balance also had a higher maximum gait speed (>160 cm/sec) during the trials on uncovered ice. On the tracks with snow-covered ice, participants could walk at a higher speed with 11 of the devices. Nine of these (#4, #8, #11–15, #17, and #19) also received ratings for good balance and low fall risk. 

**Figure 6:**
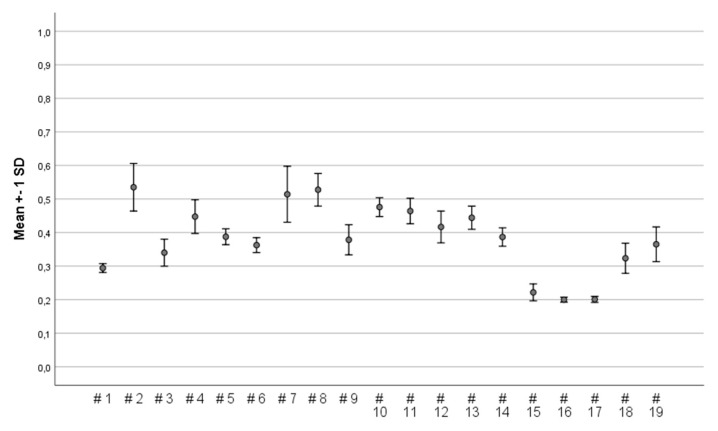
Results of DCOF for the 19 anti-slip devices. Scale 0–1.00.

The DCOF of the devices varied between 0.20 and 0.54 (scale 0−1.00). The device with the highest DCOF was device #2, designed with two studs under the front part of the shoe attached by a rubber band around the forefoot. In addition, whole-foot devices #7 and #8 (both whole-foot devices with 10–12 studs) also had high DCOF. Conversely, the least DCOF was found for three whole-foot devices designed with sandpaper (device #15), ropes (device #16), and ropes/springs (device #17; see Figure 6). 

There was a low correspondence when comparing the DCOF with participant ratings, as only one (#7) of the three devices with the highest DCOF was perceived to offer good walking balance and low fall risk. Also, for the three devices with the lowest DCOF, there was a lack of correspondence between DCOF and subjective ratings of fall risk and balance. Despite low DCOF, device #15 was perceived as providing good balance and low fall risk.

## 4. Discussion

### 4.1. Pedestrians Ratings of Fall Risk, Balance, and Footfall Transitions on Ice with Different Anti-Slip Devices 

With a few exceptions, there was little consensus between the participants in their perceptions of fall risk, balance, heel strike, or toe off when walking on uncovered ice with the different devices. This can be explained by the variation in the background characteristics due to the selection of the test group to represent a normal population of pedestrians. They differed in age, experiences, and perceptions, as well as balance abilities and their usual movement/gait patterns including step length, footfall transitions (toe/heel), or preferred walking speed. It is not surprising that people may need devices with different designs. However, of 19 tested devices, the participants agreed on three whole-foot devices and one built-in device, which they all felt provided both low fall risk and good walking balance on uncovered ice. Another five devices also stood out as providing low fall risk and good balance, but here, the pedestrians’ ratings varied more. One design that would fit all would be the best [28], therefore finding key design features perceived to be beneficial by a variety of pedestrians is essential. Earlier qualitative ratings of devices used across ice, snow, and dry concrete surfaces showed that the pedestrians preferred devices with a high number of short friction points well distributed across the sole. Factors such as safe foothold, enabling a normal gait, and predictability were also forwarded as essential for use on all surfaces [22]. 

### 4.2. Gait Speed, DCOF, and the Relations of User Ratings to these Measures 

The correspondence between the laboratory measures of DCOF and the participants’ perceptions of walking safety or risk of falling with the devices on uncovered ice was low. Only one (#7, whole foot) of the devices with the highest DCOF also offered low fall risk and walking balance. In contrast, another device (#15, sandpaper) provided good balance and low fall risk on ice, despite low DCOF. 

In general, the pedestrians’ comfortable and maximum gait speed decreased during the trials on the ice tracks. Nevertheless, devices viewed as providing low fall risk/good balance also generated the highest maximum gait speeds. Earlier research has shown that self-selected pace and step length are two independent variables that increase walking stability and reduce the risk of slipping [29]. Age-related increase in step variability and decline in one’s usual walking speed are reported [26]. When needed, most healthy older people can adopt a faster speed sufficient to cross urban streets in summer conditions. A higher speed can also induce more variable gait in terms of step length and time variability [30]. There are indications that higher gait variability may increase the risk of falls [31]. Pedestrians increasing their gait speed by increased stride length have a shorter time of double-support during their gait cycle, which places higher demands on pedestrians’ intrinsic systems to regain postural control during gait and unexpected perturbations such as when slipping. It also challenges the effectiveness of anti-slip devices to provide friction between underfoot and the support surface and in forming the base of support [22]. It is plausible that the location, height, and stability of spikes can reduce the area of the base of support, which is the point of contact the device has with the supporting surface. This could explain the low correspondence between DCOF and perceived balance and fall risk in this experiment. Earlier observations have shown that different compensatory mechanisms, such as knee flexion, shorter steps, and footfall transitions, were used to obtain balance when walking on slippery surfaces with anti-slip devices [32]. The need to visually control balance and gait by watching where they are placing their feet may also take the attention away from surrounding traffic conditions [33,34]. This is important to note, because winter conditions add the challenges of changing support surfaces and inclinations of ice or snow on pedestrian walkways and crossings [6]. To safely cross an urban street, speed, walking safety/balance, and attention are needed. 

### 4.3. Methodological Considerations

The static position with the anti-slip device sliding parallel to the ice during experimental measurements of DCOF does not reflect the rolling foothold transition during usual gait. This could explain why the present study showed no associations when comparing measures of the DCOF of the device with subjective ratings and speed. Nonetheless, for one-fifth of the devices, the pedestrians agreed in their perceptions of good balance and low fall risk, and the related measures of higher gait speed on uncovered ice.

There is, therefore, an indication that a standardised test method for classification of anti-slip devices has to a high degree be based on subjective ratings and observations of compensatory mechanisms to obtain walking safety, speed, and balance control. To be included in a standard, the measuring of DCOF has to be changed to better simulate the change in friction properties through a normal rollover from heel-strike to toe-off instead as it is now measured in a linear process with the device in a fixed position.

The test conditions maximum gait speed and uncovered ice distinguished devices providing high speed/balance and low fall risk. There was also a conformity in the pedestrians’ ratings. However, when the ice was covered with a thin layer of snow, the results differed, which indicates a need to test devices on a variety of walkway qualities encountered in urban environments. 

A strength of the study design is that the participants in this experimental study represented a normal population of pedestrians. They performed extensive trials indoors and on four different winter walkway surfaces with 19 anti-slip devices and answered qualitative surveys, hence providing multidimensional data. Repeated measurements were made on the same individuals, and data were presented mainly descriptively. Studies with an increased number of subjects and the use of random effects models to estimate the various parameters for each performance variable are needed to verify these results. 

## 5. Conclusions

In general, there was little consensus among pedestrians regarding perceptions of fall risk, balance, heel strike, or toe off using the different devices on icy pedestrian crossings. There was agreement on good balance/low fall risk for one-fifth of the tested devices (three whole-foot designs and one design with built-in spikes). The ratings for good balance/low fall risk corresponded with higher maximum gait speed, but not with DCOF. 

These findings imply that measures to increase the DCOF also need to account for the distribution of the friction points under the sole to ensure speed and walking safety/balance. To safely cross an urban street, both sufficient speed and perceived walking safety/balance are required. Methods to evaluate anti-slip devices has to reflect the rolling foothold, with its transition and forces, during gait. Especially when speeding up, the forces placed on devices through longer strides and shorter double-support phases increases the need for friction at heel strike and toe off. Also, the balance enabling properties of the device has to be satisfying, for the pedestrian to be able to walk fast with maintained balance control and perceived safety when crossing a street.

## Figures and Tables

**Table 1:** Description of the devices from the four design groups: Forefoot device (F), heel device (H), whole foot device (W), and shoes with built-in studs.

Device	Type/Spikes	Description
#1	F/5	Designed with three and two metal studs, respectively, in two rows on a metal plate under the front part of the shoe. Attached by one elastic band around the forefoot and one around the heel.
#2	F/2	Designed with two studs under the front part of the shoe attached by an elastic band around the forefoot.
#3	F/5	Designed with five studs on a rubber plate under the forefoot attached by one elastic band around the forefoot and one around the heel.
#4	H/4	A heel device designed to cover only the rear and back of the sole, attached by a Velcro band. Four studs attached on a hard rubber plate under the heel.
#5	H/4or5	Similar to device #4, with 4 (medium size) or 5 (large size) studs, attached by a Velcro band.
#6	H/5	Similar to device #4, with five studs attached by a Velcro band.
#7	W/8+4	Designed as a hard rubber plate with eight studs under the forefoot and four under the heel, attached by one elastic band over toes and one behind the heel.
#8	W6+4	A whole foot device with six studs under the forefoot and four under the heel, attached with two elastic bands over toes and one behind the heel.
#9	W/5+2	Designed with five studs under the forefoot and two under the heel, attached by one elastic band over the toes and one behind the heel.
#10	W/4+4	Designed with four studs under the forefoot and four studs under the heel.
#11	W/4+2	Designed with four studs under the forefoot and two studs under the heel.
#12	W/4+2	Whole foot device with four studs under the forefoot and two studs under the heel.
#13	W/4+2	Designed with four studs under the forefoot and two under the heel.
#14	W/4+2	Whole foot device with four studs under forefoot and two under the heel.
#15	W/sand	Whole foot device designed with one area a sand surface under forefoot and heel, respectively, framed by rubber.
#16	W/rope	Designed with a net of synthetic ropes (and twelve small metal rings on the ropes) covering the whole underfoot surface, from toe to behind the heel.
#17	W/spring	Designed with a net of synthetic ropes covered by springs under the whole underfoot.
#18	Built-in	Fixed and foldable solution with 20 studs, attached to the shoe
#19	Built-in	A solution with 16 retracting studs attached to the sole

**Table 2:** Relevant gait parameters of the subjects tested.

Test	Median (Min–Max)
Comfortable walking speed (cm/sek) ^1^	139.3 (130.7–196.1)
Maximum walking speed (cm/sek) ^1^	192.3 (158.5–243.3)
Comfortable walking speed (cm/sec) ^2^	128.8 (112.4−156.4)
Maximum walking speed (cm/sec) ^2^	183.7 (149.8−239.7)
Stride length at comfortable walking speed (cm) ^2^	78.1 (69.5−86.4)
Stride length at maximum walking speed (cm) ^2^	88.6 (79.4−104.1)
Double-support phase at comfortable walking speed (% of cycle) ^2^	23.3 (19.4−30.56)
Double-support phase at maximum walking speed (% of cycle) ^2^	19.6 (13.7−22.5)

^1^ 10 metre concrete floor, with shoes. ^2^ GAITRite® carpet, 4 metres, with shoes.

**Table 3:** Results of pedestrian gait speed and ratings of gait qualities during a walk on the indoor test track the with plain ice surface with 19 anti-slip devices. Values are median (min-max).

Ice Uncovered								
*#*	Comfortable Gait Speed ^1^	Maximum Gait Speed ^1^	Balance ^2^	Fall Risk ^2^	Heel Strike ^2^	Toe Off ^2^	*p* 3	*p* 4
1	140.2 (112.1−172.1)	146.2 (104.0−198.4)	71 (20−99)	36 (1−97)	35 (5−99)	78 (49−99)	0.374	0.011
2	132.1 (101.2−142.9)	147.5 (116.9−181.8)	66 (30−100)	43 (7−59)	21 (3−68)	57 (13−100)	0.036	0.011
3	136.2 (122.0−150.2)	151.1 (114.2−181.5)	78 (25−100)	27 (1−95)	31 (1−99)	71 (35−99)	0.173	0.011
4	138.7 (119.9−142.9)	146.2 (142.2−168.6)	*88 (58−98)*	*20 (0−91)*	91 (15−99)	51 (5−98)	0.441	0.008
5	129.2 (124.7−151.5)	153.1 (136.4−175.1)	70 (50−98)	23 (3−82)	80 (73−99)	23 (8−99)	0.066	0.008
6	133.3 (113.9−153.1)	154.6 (103.6−175.4)	74 (41−96)	*19 (0−62)*	71 (54−95)	25 (6−85)	0.110	0.008
7	136.4 (117.6−163.4)	**165.8** (123.3−183.8)	*83 (52−98)*	*3 (2−53)*	75 (45−98)	80 (48−99)	0.173	0.008
8	129.9 (110.7−144.9)	143.3 (116.7−175.8)	*81 (22−92)*	31 (0−70)	62 (39−94)	65 (38−91)	0.028	0.008
9	129.2 (111.5−143.5)	144.3 (111.7−179.5)	71 (24−98)	*16 (2−65)*	55 (33−95)	58 (31−95)	0.021	0.011
10	121.6 (110.4−173.0)	145.6 (96.3−196.5)	67 (45−99)	38 (4−71)	58 (28−94)	62 (28−90)	0.260	0.011
11	140.4 (114.2−157.2)	**164.7** (132.4−181.8)	*93 (64−99)*	*10 (1−65)*	76 (1−99)	89 (6−98)	0.678	0.008
12	127.7 (119.8−144.7)	154.6 (118.6−174.2)	*81 (65−100)*	*19 (0−54)*	70 (55−100)	71 (50−100)	0.028	0.008
13	129.9 (122.6−154.6)	**176.7** (122.0−194.6)	***88 (80−100)***	***11 (0−23)***	80 (63−100)	79 (68−100)	0.051	0.028
14	138.0 (125.9−155.3)	**164.5** (127.9−183.8)	***93 (76−100)***	***6 (1−29)***	86 (71−99)	90 (63−99)	0.314	0.011
15	139.7 (125.3−153.8)	**162.5** (129.5−194.2)	***90 (78−100)***	***5 (0−23)***	77 (68−100)	78 (49−97)	0.069	0.025
16	123.2 (105.8−145.6)	151.5 102.2−171.5)	42 (17−98)	73 (1−99)	55 (1−95)	56 (2−95)	0.015	0.008
17	134.4 (99.4−157.5)	158.5 (107.9−177.6)	72 (24−100)	26 (7−85)	65 (28−100)	61 (22−95)	0.021	0.008
18	131.6 (107.8−197.6)	159.5 (103.5−203.7)	*82 (29−96)*	*12 (2−80)*	56 (6−95)	56 (12−96)	0.139	0.008
19	138.5 (118.9−158.2)	**166.7** (130.4−188.7)	***96 (82−100)***	***3 (0−11)***	95 (73−100)	96 (72−100)	0.214	0.011

^1^ Cm/sec. Bold digits denote a ‘higher maximum gait speed’ ≥160 cm/sec. ^2^ Scale 0–100. *Italic* digits denote a ‘good walking balance’ ≥80 points, ‘low fall risk’ ≤20 points. Italic & bold digits denote ‘agreement’, that is, inter-subject variations ≤30 points. *p*-values compares repeated measurements in the same sample, and denotes the difference between gait speed at baseline without devices and comfortable gait speed (*p* 3) and maximum speed on ice (*p* 4) respectively, and are based on the Wilcoxon signed ranks test.

**Table 4:** Results of pedestrian gait speed and ratings of gait qualities during walk on the indoor test track with snow-covered ice surface with 19 anti-slip devices. Values are median (min-max).

Snow-Covered Ice								
#	Comfortable Gait Speed ^1^	Maximum Gait Speed ^1^	Balance ^2^	Fall Risk ^2^	Heel Strike^2^	Toe Off ^2^	*p* 3	*p* 4
1	148.1 (101.7−176.7)	155.8 (99.8−205.3)	68 (27−92)	32 (3−59)	40 (4−78)	60 (30−95)	0.314	0.011
2	132.3 (117.5−151.8)	149.9 (110.5−182.2)	62 (32–94)	44 (0−76)	20 (3−65)	52 (4−74)	0.038	0.008
3	135.7 (121.1−157.7)	**160.0** (131.8−191.6)	68 (5−98)	22 (3−100)	35 (2−99)	67 (40−98)	0.139	0.008
4	142.8 (129.0−177.9)	**167.5** (131.2−193.05)	*92 (64−98)*	*14 (2−66)*	79 (11−99)	31 (16−98)	0.953	0.011
5	135.1 (128.7−153.8)	155.0 (126.3−180.2)	78 (47−98)	23 (2−80)	80 (56−97)	54 (14−96)	0.214	0.011
6	143.7 (117.2−170.4)	**165.0** (123.6−177.9)	74 (60−95)	21 (0−76)	62 (42−91)	55 (13−89)	0.314	0.008
7	139.1 (113.5−173.9)	159.0 (100.8−187.6)	*82 (21−96)*	*6 (1−97)*	80 (18−95)	76 (17−98)	0.594	0.008
8	142.8 (111.9−159.2)	**165.0** (149.0−189.4)	*92 (22−98)*	*19 (2−48)*	84 (27−97)	82 (27−95)	0.767	0.015
9	138.5 (117.4−154.1)	157.7 (109.5−176.1)	74 (21−99)	*15 (3−50)*	71 (38−99)	71 (39−97)	0.260	0.008
10	133.0 (90.8−170.1)	158.2 (87.6−184.8)	61 (28−97)	25 (2−67)	60 (26−94)	63 (26−94)	0.161	0.008
11	141.4 (120.3−161.3)	**170.1** (126.1−191.9)	*90 (63−99)*	***12 (1−29)***	78 (56−98)	79 (34−98)	0.953	0.008
12	133.3 (119.1−157.5)	**160.0** (128.4−172.7)	*91 (64−99)*	*11 (0−41)*	66 (41−99)	62 (15−88)	0.051	0.008
13	136.4 (121.1−156.5)	**160.2** (130.4−176.7)	*90 (66−100)*	*11 (0−34)*	93 (64−99)	92 (8−99)	0.214	0.008
14	139.3 (127.6−164.2)	**160.0** (126.4−178.9)	***87 (70−98)***	*10 (2−59)*	91 (59−98)	83 (35−97)	0.441	0.012
15	140.8 (130.0−154.6)	**160.6** (126.3−183.2)	***88 (81−99)***	*8 (1−60)*	76 (50−100)	76 (44−91)	0.263	0.012
16	142.8 (130.6−159.0)	156.2 (127.4−198.0)	*87 (37−98)*	*9 (2−80)*	86 (7−100)	88 (11−100)	0.484	0.008
17	141.0 (116.0−159.0)	**167.5** (109.3−190.8)	*83 (55−100)*	*18 (0−52)*	69 (41−96)	63 (40−100)	0.441	0.008
18	137.4 (120.3−158.2)	158.2 (139.7−186.6)	*92 (48−96)*	*8 (0−75)*	84 (12−95)	82 (14−95)	0.139	0.008
19	147.1 (121.2−171.2)	**169.8** (128.4−191.2)	**96 (77−100)**	*4 (0−36)*	95 (5−100)	94 (34−100)	0.515	0.008

^1^ Cm/sec. Bold digits denote a ‘higher maximum gait speed’ ≥160 cm/sec. ^2^ Scale 0–100. *Italic* digits denote a ‘good walking balance’ ≥80 points, ‘low fall risk’ ≤20 points. Italic & bold digits denote ‘agreement’, that is, inter-subject variations ≤30 points. *p*-values compares repeated measurements in the same sample, and denotes the difference between gait speed at baseline without devices and comfortable gait speed (*p* 3) and maximum speed on ice (*p* 4) respectively, and are based on the Wilcoxon signed ranks test.
